# PRSS8 methylation and its significance in esophageal squamous cell carcinoma

**DOI:** 10.18632/oncotarget.8677

**Published:** 2016-04-11

**Authors:** Yonghua Bao, Qian Wang, Yongchen Guo, Zhiguo Chen, Kai Li, Yiqiong Yang, Huijuan Zhang, Huali Dong, Kui Shen, Wancai Yang

**Affiliations:** ^1^ Department of Pathology and Institute of Precision Medicine, Jining Medical University, Jining 272067, China; ^2^ Department of Immunology, Xinxiang Medical University, Xinxiang 453003, China; ^3^ Department of Pathology, Xinxiang Medical University, Xinxiang 453003, China; ^4^ Department of Pathology, The First Affiliated Hospital, Xinxiang Medical University, Xinxiang 453003, China; ^5^ Department of Pathology, University of Illinois at Chicago, Chicago, IL 60612, USA

**Keywords:** PRSS8, methylation, esophageal cancer, survival, cancer stroma

## Abstract

Esophageal cancer is one of the most common cancers worldwide, and the incidence and mortality is increasing rapidly in recent years in China, but the underlying mechanisms are largely unclear. Herein we found that the expression of PRSS8, a serine protease prostasin, is significantly decreased in esophageal squamous cell carcinomas (ESCC) at mRNA and protein levels. The reduction of PRSS8 was well correlated with poor differentiation and shorter survival time. Interestingly, ESCC stromal expression of PRSS8 was significantly correlated with stromal lymphocyte infiltration and cancer progression. Methylation specific PCR showed that PRSS8 was hypermethylated in ESCC tissues and ESCC cell lines, which was linked to the downregulation of PRSS8 expression and decreased activities of PRSS8 promoter. De-methylation agent decitabine was able to restore PRSS8 expression, leading to the inhibition of cancer cell proliferation, motility, migration and cell cycle arrest. However, the restored PRSS8 and its tumor inhibition could be reversed by small interfering RNA targeting PRSS8. Mechanistic study showed that tumor inhibition of PRSS8 may be associated with proliferation- and epithelial mesenchymal transition - related proteins in ESCC cells. In conclusion, our finding showed that PRSS8 methylation and its stromal expression had important clinical significance in ESCC.

## INTRODUCTION

Esophageal cancer is one of the most common cancers worldwide [1], the incidence is increasing rapidly in recent years in China, and is the fourth most frequent cause of cancer-related deaths in China, five-year survival is very poor [2]. However, the underlying mechanisms are largely unknown, although epidemiological and etiological studies have shown the crucial roles of environmental and genetic factors in esophageal carcinogenesis, resulting in more than 95% of esophageal squamous cell carcinoma (ESCC) in China and more than 90% of esophageal adenocarcinomas in the American and European [3, 4]. Numerous studies have shown that the silence or decreased expression of tumor suppressor genes could be one of the major causes of esophageal carcinogenesis, in particular, promoter hypermethylation of tumor suppressor genes leads to silence and downregulation of gene expression, which is linked to tumor formation and progression [5–8]. In the present study, we found that PRSS8 (protease serine 8), a trypsin-like serine peptidase [9–11], is hypermethylated in ESCC tissues and ESCC cell lines.

PRSS8, also known as Prostasin, was found highly expressed in normal prostate gland and seminal fluid. Further studies have demonstrated that PRSS8 is overexpressed in epithelial cells of various tissues, is involved in epithelial differentiation and shows important roles in epidermal barrier function, skin phenotypes, and embryonic viability [10, 11]. Interestingly, recent studies have reported that serum PRSS8 level is increased in ovarian cancer patients [12], but PRSS8 expression was decreased in chemoresistant ovarian cancer patients and chemoresistant cell line. Moreover, increased expression of PRSS8 induced cells death in ovarian cancer cell lines [13]. In addition, PRSS8 expression was reduced in the cancers of prostate [14, 15], breast [16], bladder [17] and stomach [18], showing tumor suppressive roles. In prostate cancer, reduced PRSS8 was associated with hypermethylation of PRSS8 [17]. However, the methylation site was not clear and whether the methylation has biological functions is unknown either. Herein we found that PRSS8 was significantly reduced in ESCC cancer tissues and cancer cells at protein and mRNA levels, and the reduction of expression was associated with poor differentiation and shorter survival time and disease-free time. More importantly, the PRSS8 methylation likely played a crucial roles in ESCC, evidenced by restoration of PRSS8 by de-methylation agent and knockdown the restoration of PRSS8 in ESCC cells exhibiting inverse functions.

## RESULTS

### PRSS8 was reduced in ESCC tissues and the reduction of PRSS8 was associated with poor differentiation and shorter survival time

**Figure 1:**
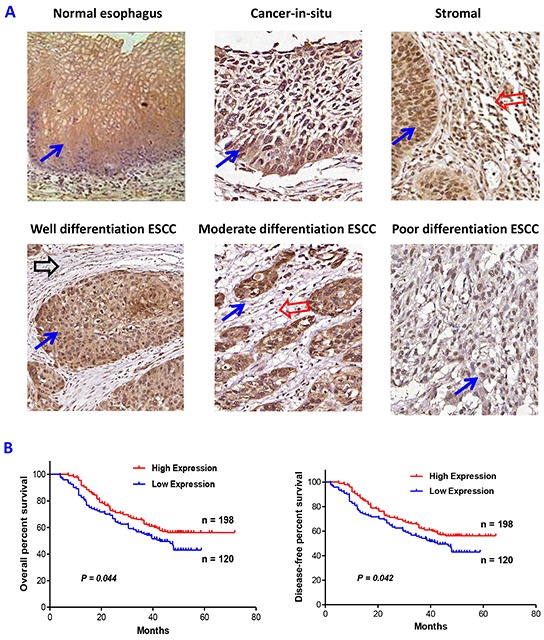
Altered expression of PRSS8 in esophageal squamous cell carcinoma (ESCC) **A.** PRSS8 expression in normal esophagus, cancer-in-situ, cancer stromal, well-differentiated, moderate-differentiated and poorly-differentiated ESCC. (Blue arrows indicated the positive epithelial staining, red arrows indicated positive stromal staining areas, black arrow indicated no or weak stromal staining area). **B.** PRSS8 expression levels in ESCC were significantly associated with overall survival time (left panel) and disease free period (right panel) of the patients with ESCC.

To determine the expression levels of PRSS8, a tissue microarray (TMA) containing 362 cases of ESCC tissues was examined using immunohistochemical staining. As shown in Figure 1A, PRSS8 was highly expressed in normal esophageal epithelia mainly in membrane and cytoplasm and partially in nuclei. Importantly, PRSS8 expressed in epithelia was greatly associated with cancer differentiation (Figure 1A and Table 1). For example, PRSS8 was overexpressed in cancer-in-situ, and in well- and moderate-differentiated ESCC tissues, but the expression was dramatically reduced in the poorly differentiated ESCC tissues, the difference was significant (p<0.001, Table 1). Moreover, 67% (12/18) of the less malignant cancers (i.e. cancer-in-situ) exhibited strong staining (score 3), but only 29% (100/344) of the invasive cancer showed strong staining (p=0.0017, Table 1). Further analysis revealed that the higher epithelial PRSS8 expression in ESCC exhibited significantly longer overall survival time (p=0.044) and disease-free period (p=0.042, Figure 1B).

**Table 1:** The correlation of PRSS8 expression in esophageal epithelia or stroma and clinicopathological characteristics

Clinicopathologic Variable	N	PRSS8 expression (epithelial cells)			*P value*	PRSS8 expression (stromal tissues)		*P value*
		3	2	1 and 0		2	1 and 0	
**Age**					0.800			0.600
≤60	158	59 (37%)	57 (36%)	42 (27%)		40 (25%)	118 (75%)	
>60	204	68 (33%)	73 (36%)	63 (31%)		70 (34%)	134 (66%)	
**Gender**					0.720			0.245
Male	230	80 (35%)	89 (39%)	61 (26%)		65 (28%)	165 (72%)	
Female	132	47 (36%)	41 (31%)	44 (33%)		45 (34%)	87 (66%)	
**Smoking**					0.685			0.560
Yes	154	62 (40%)	51 (33%)	41 (27%)		48 (31%)	106 (69%)	
No	208	75 (36%)	71 (34%)	62 (30%)		59 (28%)	149 (72%)	
**Drinking**					0.327			0.670
Yes	100	38 (38%)	39 (39%)	23 (23%)		29 (29%)	71 (71%)	
No	262	91 (35%)	90 (34%)	81 (31%)		82 (31%)	180 (69%)	
**Lymphatic metastasis**					0.624			0.973
Yes	161	59 (37%)	59 (37%)	43 (26%)		51 (32%)	110 (68%)	
No	201	68 (34%)	70 (35%)	63 (31%)		64 (32%)	137 (68%)	
**Lymphocyte infiltration**								**0.001**
Yes	207					**77 (37%)**	130 (63%)	
No	155					33 (21%)	122 (79%)	
**Differentiation/malignancy**					**<0.001**			
Well/moderate	235	98 (42%)	79 (34%)	58 (24%)				
Poor	127	30 (23%)	44 (35%)	53 (42%)				
					**0.0017**			**0.017**
*Cancer in situ*	18	12 (67%)	5 (28%)	1 (5%)		10 (55%)	8 (45%)	
Invasive cancer	344	100 (29%)	118 (34%)	126 (37%)		100 (29%)	244 (71%)	

Moreover, ESCC stroma was also found positively stained (Figure 1A), although the staining intensity was not as strong as observed in the epithelia. Interestingly, cancer stromal staining of PRSS8 was well correlated with stromal lymphocyte infiltration, in which, 37% (77/207) of cancer stroma showed higher PRSS8 expression if the stroma presented visible lymphocyte infiltration, whereas, only 21% (33/155) of the stroma showed higher expression of PRSS8 if the stroma did not present visible lymphocyte infiltration, the difference was statistically significant (p=0.001, Table 1). In addition, the stroma of cancer in situ showed more expression of PRSS8 compared to that in invasive ESCC stroma (p=0.017, Table 1). It is worthy to be noted that the positive correlation of stromal expression of PRSS8 and lymphocyte infiltration could be resulted from a feedback or response, suggesting that PRSS8 acts as a protector or suppressor to prevent cancer invasion and progression. Actually, more lymphocyte infiltration in the stroma is associated with better outcomes of the ESCC patients (Bao, et al, unpublished data).

We then determined the mRNA levels of PRSS8 in ESCC tissues using available online data sets and found that PRSS8 mRNA levels were also dramatically reduced in ESCC tissues in comparison with their adjacent normal esophagus, evidenced by mining two sets of gene profile data (Supplementary Figure S1, left panel (GDS3838) [19] and middle panel from Oncomine [20]). Moreover, PRSS8 mRNA levels were also reduced in Barrett's Esophagus (BE), a precancerous lesion of esophageal adenocarcinoma (EAC), and were reduced further in EAC (Supplementary Figure S1, right panel from Oncomine [21]).

### PRSS8 was differentially expressed in ESCC cell lines, and the reduction of PRSS8 was associated with PRSS8 promoter hypermethylation

**Figure 2:**
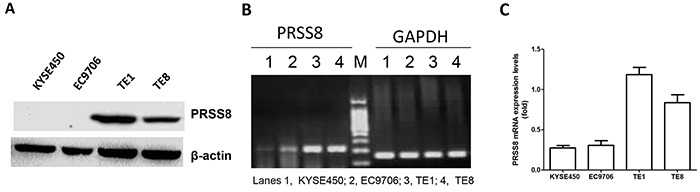
Differential expression of PRSS8 in esophageal cancer cell lines **A.** PRESS8 protein levels in ESCC cell lines (KYSE450, EC9706, TE1 and TE8), assayed by immunoblotting. **B.** semi-quantification of PRSS8 mRNA in ESCC cell lines by RT-PCR. GAPDH was used as internal control. **C.** PRSS8 mRNA levels in ESCC cell lines by quantitative RT-PCR. The quantity of PRSS8 mRNA levels was neutralized to GAPDH.

We then determined PRSS8 expression levels in ESCC cell lines. Four human ESCC cell lines (KYSE450, EC9706, TE1 and TE8) were screened for PRSS8 expression. Immunoblotting results showed that PRSS8 protein levels were expressed in TE1 and TE8 cells, but were almost undetectable in KYSE450 and EC9706 cells (Figure 2A). The mRNA levels of PRSS8 in the four cell lines were similar as the protein levels assayed by semi-RT-PCR (Figure 2B) and quantitative RT-PCR (Figure 2C).

**Figure 3:**
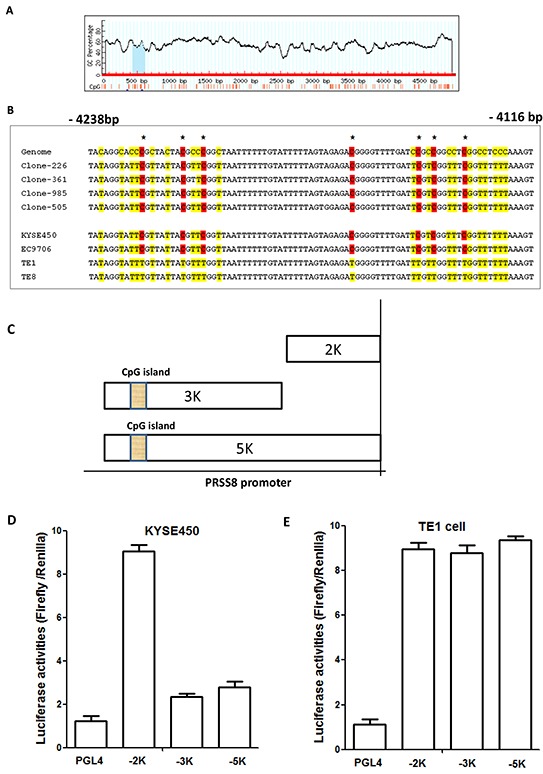
Reduced expression of PRSS8 was associated with hypermethylation in PRSS8 promoter region **A.** A CpG island was identified in PRSS8 promoter region (−4116 to - 4238). **B.** Methylation specific PCR and PCR product sequence showed hypermethylation in 4 esophageal squamous cell carcinomas (Clones-226, -361, -985 and -505) and in 2 esophageal cancer cell lines (KYSE450, EC9706), other 2 esophageal cancer cell lines (TE1 and TE8) did not show hypermethylation. **C.** PRSS8 promoter truncated mutations were constructed into pGL4 vector, respectively. **D.** and **E.** PRSS8 truncated mutations showed different activities in HEK293 cells, suggesting that methylated promoter from KYSE450 cells impaired reporter activity, compared to the unmethylated promoter from the TE1 cells. The experiments were conducted three times independently.

To determine whether the reduction of PRSS8 in ESCC tissues and cell lines was caused by hypermethylation in PRSS8 promoter region, we used the methylation CpG island prediction software [22] and identified one CpG island in PRSS8 promoter region (−4238 to -4116) (Figure 3A). Methylation specific PCR (MS-PCR) was then used to amplify this region of some ESCC tissues and the four ESCC cell lines. Figure 3B showed the examples of PRSS8 promoter region hypermethylation in ESCC tissues (4 clones) and in 2 ESCC cell lines (KYSE450 and EC9706) that showed undetectable PRSS8 level, but another 2 cell lines (TE1 and TE8) exhibited expression of PRSS8 and showed no methylation in PRSS 8 promoter region.

To determine whether this CpG island at the PRSS8 promoter region has biological function, we then constructed reporters with truncated PRSS8 promoter region into pGL4 vector (Promega, Madison, WI). As shown in Figure 3C, a full length of PRSS8 promoter (5000bp, 5K), a 3000bp (3K) and a 2000bp (2K) region, from KYSE450 cell (This cell line has hypermethylated promoter) and TE1 cells (This cell line does not have a hypermethylated promoter region), were respectively constructed. These constructs and negative control pGL4 vector were transfected into human embryonic kidney cells (HEK293 cells), respectively, with co-transfection of internal control Renilla. As shown in Figure 3D and 3E, truncated PRSS8 promoter reporters with hypermethylated and unmethylated promoter regions showed different activities. Unmethylated 2K reporter from both cell lines showed the strongest activities, but methylated 3K and 5K reporters from the KYSE450 cells showed lower activities (Figure 3D). However, the unmethylated 3K and 5K reporters showed similar higher activities as 2K reporter (Figure 3E). These findings indicated that PRSS8 promoter methylation in KYSE450 cells significantly impacted promoter reporter activity, compared to TE1 cells that have no methylation, suggested that the CpG island in PRSS8 promoter region indeed has biological functions, and this region hypermethylation might lead to repression of PRSS8 expression.

### PRSS8 expression could be restored by demethylation agent decitabine, and the restoration of PRSS8 could be reversed by small interfering RNA in ESCC cells

**Figure 4:**
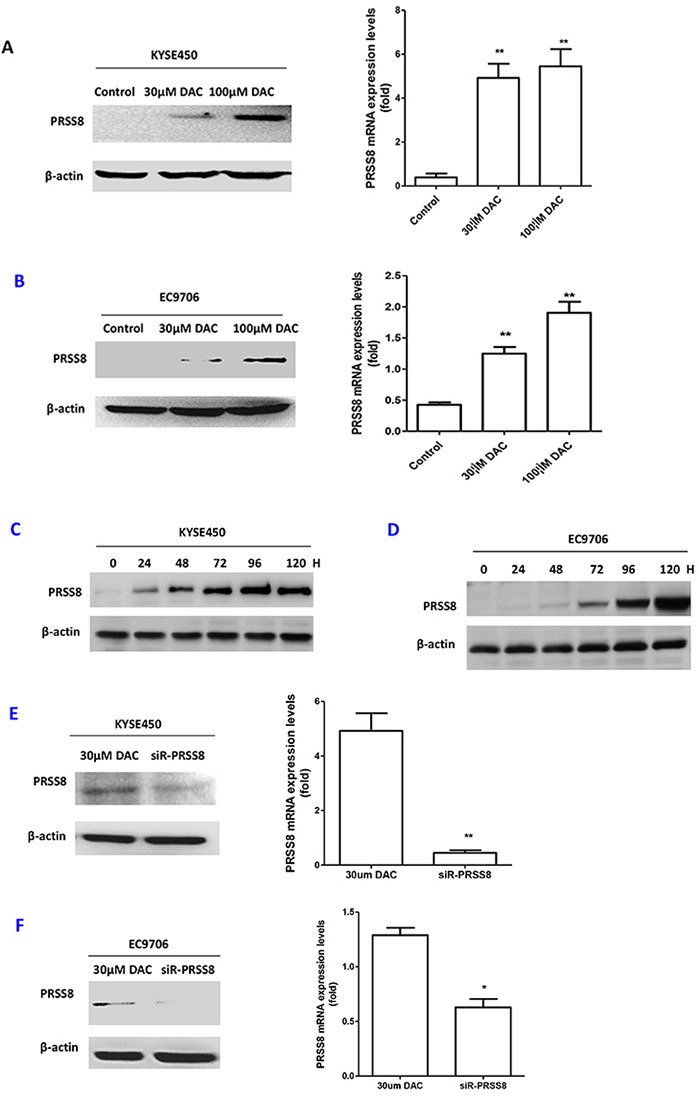
PRSS8 expression levels were restored by de-methylation agent decitabine (DAC), and the restored expression could be reduced by small interfering RNA targeting human PRSS8 (siR-PRSS8) **A.** PRSS8 expression levels were restored by DAC at protein and mRNA levels in KYSE450 cells. **B.** Similar results were seen in another ESCC cell line EC9706. **C** and **D.** the restoration of PRSS8 by 100μM of DAC was time-dependent in KYSE450 (C) and EC9706 cells (D). (H stood for hours). **E** and **F.** Small interfering RNA targeting PRSS8 attenuated DAC-induced PRSS8 expression in ESCC cell line KYSE450 (E) and EC9706 cells (F). **p<0.01, *p<0.05, compared to the control group. All experiments were triplicated independently.

5-Aza-2′-deoxycytidine (Decitabine, DAC) is an inhibitor of DNA methyltransferase and has been widely used as a demethylation agent [23]. Therefore, we treated the KYSE450 and EC9706 cells with 30 μM or 100 μM of DAC, respectively, as we reported previously [24]. As expected, 30μM and 100μM of DAC were able to restore PRSS8 expression at protein and mRNA levels in KYSE450 (Figure 4A) and EC9706 cells (Figure 4B), assayed by immunoblotting and quantitative RT-PCR. To determine whether the restoration of PRSS8 by DAC was time-dependent, we treat KYSE450 and EC9706 cells with DAC for 24, 48, 72, 96 and 120 hours. As shown in Figure 4C and 4D, DAC-induced PRSS8 expression was time-dependent. Moreover, we treated the DAC-treated cells with small interfering RNA targeting human PRSS8 (siR-PRSS8) and found that the restoration of PRSS8 could be knocked down by siR-PRSS8 at protein and mRNA levels in KYSE450 (Figure 4E) and EC9706 cells (Figure 4F).

### DAC led to the inhibition of cell proliferation, motility, migration and cell cycle arrest at G1 phase, which was reversed by siR-PRSS8

**Figure 5:**
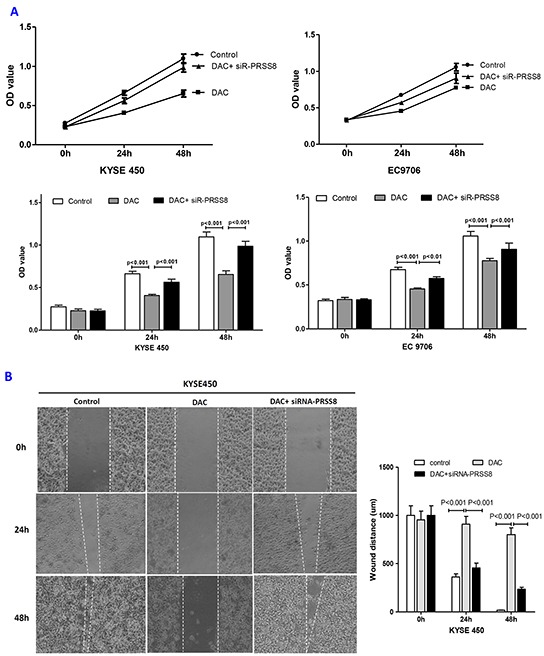

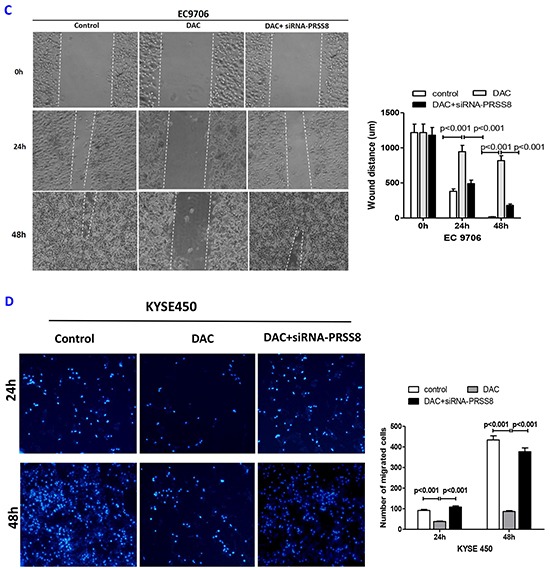

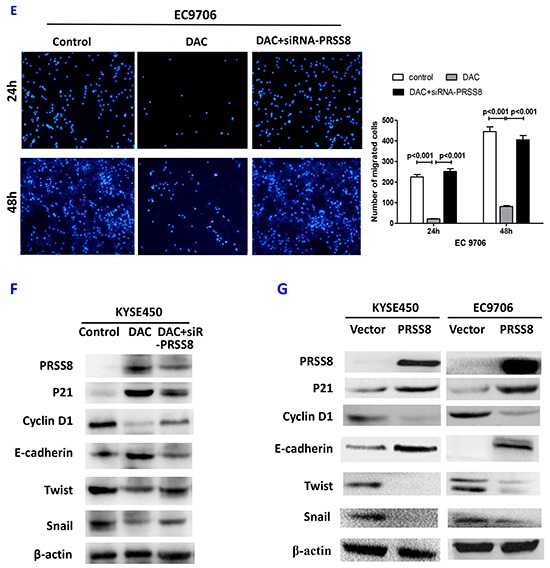
Restored PRSS8 expression by DAC led to inhibition of cell proliferation, motility and migration, but PRSS8-mediated tumor inhibition could be attenuated by small interfering RNA **A.** Restored expression of PRSS8 inhibited cell proliferation in KYSE450 and EC9706 cells, but the inhibition was reversed by siRNA targeting PRSS8 (siR-PRSS8), assayed by MTT. The statistical analysis was shown in the lower panel. **B** and **C.** Restored PRSS8 inhibited cell motility in KYSE450 cells (B) and EC9706 cells (C), but the inhibition was reversed by siRNA, assayed by wound healing. The quantification of the wound width was shown in the right panel, respectively. **C.** Restored PRSS8 inhibited cell motility in KYSE450 cells (B) and EC9706 cells (C), but the inhibition was reversed by siRNA, assayed by wound healing. The quantification of the wound width was shown in the right panel, respectively. **D.** Restored expression of PRSS8 inhibited cell migration in KYSE450 (D) and EC9706 cells (E), but the inhibition was reversed by siRNA. The number of the migrated cells was shown in the right panel, respectively. **E.** Restored expression of PRSS8 inhibited cell migration in KYSE450 (D) and EC9706 cells (E), but the inhibition was reversed by siRNA. The number of the migrated cells was shown in the right panel, respectively. **F.** The alteration of proliferation and EMT-related proteins by DAC and DAC + siR-PRSS8 in KYSE450 cells was shown. **G.** PRSS8 overexpression led to the upregulation of P21 and E-cadherin and to the downregulation of cyclin D1, Twist and Snail in KYSE450 and EC9706 cells.

To determine the biological roles of PRSS8 in ESCC cells, particularly the significance of PRSS8 promoter methylation, we treated KYSE450 and EC9706 cells with 100μM DAC or DAC+siR-PRSS8 for 24 and 48 hours, and determined cell proliferation, motility and migration using MTT, wound healing and transwell methods. We found that DAC significantly inhibited ESCC cell proliferation (Figure 5A), motility (Figure 5B and 5C) and migration (Figure 5D and 5E). Interestingly, tumor inhibition by DAC-mediated restoration of PRSS8 was reversed by small interfering RNA targeting PRSS8, for instance, cell proliferation, motility and migration in the DAC+siR-PRSS8 groups were similar as in the control groups in KYSE450 and EC9706 cells (Figures 5A–5E). Taken above, these findings strongly suggested that PRSS8 has tumor suppression functions and PRSS8 promoter hypermethylation has important significance.

In addition, restoration of PRSS8 led to cell cycle arrest at G1 phase in both KYSE450 and EC9706 cell lines, but this G1 phase arrest was attenuated by small interfering RNA, in comparison to the control groups (Table 2).

**Table 2:** DAC-induced PRSS8 expression led to cell cycle arrest at G1 phase in ESCC cell lines KYSE450 and EC9706, which was reversed by siRNA knockdown

	KYSE450			EC9706		
	Control	DAC	DAC+ siRNA	Control	DAC	DAC+siRNA
**G1 phase**	25.1%	50.7%*	27.3%	30.8%	58.2%*	29.3%
**G2 phase**	32.1%	39.6%	31.9%	38.0%	28.0%	37.0%
**S phase**	42.8%	9.7%*	40.8%	31.2%	13.8%*	33.7%
**Total**	100%	100%	100%	100%	100%	100%

Note:

* *p<0.05*, compared to the control group (DAC stood for decitabine;

siRNA stood for the siRNA targeting human PRSS8).

### Mechanisms of tumor inhibition by PRSS8

Since PRSS8 exerts tumor inhibition functions in ESCC cells, we then determined the potent mechanism. To do so, we firstly determined the alteration of cell proliferation and epithelial-mesenchymal transition (EMT)-related proteins. As shown in Figure 5F, DAC induced PRSS8 expression, upregulated P21 and E-cadherin expression, and downregulated the expression of Cyclin D1, Twist and Snail. However, the alteration was reversed by siRNA. We then transfected PRSS8 overexpression plasmid (pEGFP -PRSS8) into KYSE450 and EC9706 cells. The cells were collected 48 hours after transfection, and the cell lysate was used for immunoblotting. As shown in Figure 5G, overexpression of PRSS8 led to the alterations of cell proliferation-related proteins (i.e. increase of P21WAF1 and decrease of Cyclin D1), and led to the alterations of epithelial-mesenchymal transition (EMT)-related proteins (e.g. upregulation of E-cadherin and downregulation of Twist and Snail) [25] in KYSE450 and EC9706 cells.

## DISCUSSION

Numerous studies have revealed that epigenetic alterations play a pathological role in cancer initiation and progression [26–28]. DNA methylation is an enzymatic process involving the addition of a methyl group to the 5′-position of the pyrimidine ring of cytosines to produce 5-methylcytosine. This covalent modification is catalysed by DNA methyltransferases (DNMTs) in short CpG-rich DNA stretches known as CpG islands. CpG islands overlap the promoter region and the promoters may become aberrantly hypermethylated, leading to transcriptional repression, in terms of reduction of gene expression [29]. Indeed, promoter hypermethylation-induced inactivation of tumor suppressor genes has been observed at the multistep process of carcinogenesis [30]. Esophageal cancer is one of the most common cancers, and Northern China areas, particularly, the Linxian City, has the highest incidence of esophageal squamous cell carcinoma in the world. In this study, we have found that PRSS8 promoter was hypermethylated in esophageal squamous cell carcinoma tissues and cancer cell lines, resulting in downregulation of PRSS8 at mRNA and protein levels, which was supported by the GEO Profiles (http://www.ncbi.nlm.nih.gov/geoprofiles/) and Oncomine (http://www.oncomine.com) online data (Supplemental Figure 1) showing that PRSS8 was significantly reduced in esophageal squamous cell carcinoma and adenocarcinoma in comparison with the adjacent non-tumor esophagus.

The biological functions and the significance of PRSS8 promoter methylation was supported by the following observations: first, reduced expression of PRSS8 was linked to promoter methylation, and promoter truncation reporters confirmed the role of the CpG island in the promoter region; second, DNA methyltransferase inhibitor DAC was able to restore PRSS8 expression, exhibiting tumor inhibition functions, such as the inhibition of cancer cell proliferations, mobility, migration and cell cycle arrest at G1 phase, in contrast, the small interfering RNA knocking down DAC-mediated PRSS8 expression attenuated the functions of tumor inhibition. As addressed above, the cell growth was measured by MTS assay (Figure 5A) and could be reflected by cell mobility assayed with wound healing (Figure 5B and 5C), in another word, cell mobility could be the effect of cell growth, proliferation and motility. Whether the restoration of PRSS8 expression by DAC could occur in vivo and whether DAC could be used as a potential agent for esophageal cancer therapy need to be firstly tested in animal model. This hypothesis is under investigation in carcinogen N-nitrosomethylbenzylamine (NMBA)-induced esophageal mouse model.

Previous studies have reported that the inactivation of tumor suppressor genes was one of the major causes of esophageal carcinogenesis, and hypermethylation-induced silence of tumor suppressors, such as P16 [31, 32], E-cadherin [27] and selenium-binding protein 1 [33], etc, are frequently observed in esophageal cancers. However, these studies have only revealed the association and none of them has clearly shown the important role of hypermethylation in esophageal cancer. We used the approaches of restoration by demethylation, truncated promoter reporters, and knockdown by small interfering RNA, to provide direct evidence identifying the critical role of PRSS8 in esophageal cancer. More interestingly, the alterations of PRSS8 expression were well correlated with esophageal cancer differentiation and outcomes of the ESCC patients, indicating that PRSS8 might be a useful biomarker for the evaluation of ESCC differentiation and for the prediction of ESCC outcomes.

Most importantly, ESCC stromal expression levels of PRSS8 was significantly correlated with stromal lymphocyte infiltration and cancer progression. We found that besides epithelial expression of PRSS8 in ESCC, PRSS8 was also expressed in ESCC stromal tissues, and stromal expression levels were positively correlated with inflammatory cell infiltration. This is the first to be reported. Recent evidences have shown that the interaction between epithelia and stroma plays crucial roles in facilitating cancer cell invasion and metastasis, and the inflammatory cells in the cancer areas plays protection roles [34]. Thus, the positive correlation of PRSS8 expression and lymphocyte infiltration in the ESCC stroma could be resulted from a feedback or response to prevent cancer cell invasion and limit cancer metastasis, therefore these findings provides additional evidence of tumor suppression role of PRSS8.

Tumor suppressor roles of PRSS8 were supported by *in vitro* study by transfection of PRSS8 overexpressing plasmid, showing that increased expression of PRSS8 led to the upregulation of P21 and E-cadherin and to the downregulation of Cyclin D1, Snail and Twist, which are well associated with cell proliferation and epithelial mesenchymal transition.

In conclusion, we have identified that PRSS8 acts as a tumor suppressor gene in ESCC, the hypermethylation of the promoter region leads to repression of expression, and reduced expression is significantly associated with cancer differentiation and survival. Most interestingly, the ESCC stromal expression of PRSS8 is positively correlated with stromal inflammatory cell infiltration, suggesting a preventive role of PRSS8 in the stroma. Taken together, our findings have demonstrated that PRSS8 methylation and its stromal expression has important clinical significance in esophageal squamous cell carcinoma.

## MATERIALS AND METHODS

### The data of human esophageal cancer PRSS8 mRNA expression level

The data sets used for analyzing of human esophageal cancer PRSS8 mRNA expression levels were obtained from the GEO Profiles (http://www.ncbi.nlm.nih.gov/geoprofiles/), and Oncomine (http://www.oncomine.com). The data from more research groups ensured the accuracy of the data analysis results.

### Human esophageal cancer samples and tissue microarray (TMA)

Human esophageal tissues were obtained from the Tissue Bank of the Laboratory for Cancer Signaling Transduction at Xinxiang Medical University, and from the Institute of Precision Medicine of Jining Medical University, China. The human esophageal squamous cell carcinoma tissue microarray (TMA) with survival information was made in our laboratory. All procedures were approved by the Institutional Review Board of Xinxiang Medical University and the Institutional Review Board of Jining Medical University.

### Immunohistochemical staining, staining intensity evaluation and survival analysis

The ESCC TMA blocks were sectioned, de-paraffinned and rehydrated, then treated with 3% H_2_O_2_, and then incubated with primary antibodies and biotinylated secondary antibodies. The immune complexes were visualized using the Strept Avidin-Biotin Complex kit (Boster Biological Tech. LTD., Wuhan, China). The staining intensities were scored as: 0 and 1, no/low staining (absent or weak staining); 2, moderate staining; 3, high staining (strong staining). Cancer patient survival analysis was performed using Kaplan-Meier method and GraphPad Prism 5.0 software (La Jolla, CA).

### Quantitative reverse-transcriptional polymerase chain reaction (qRT-PCR)

Total RNA was extracted from the ESCC cells using Trizol reagent (Invitrogen, Carlsbad, CA) following the manufacturer's protocol. qRT-PCR (Applied Biosystem Inc.) was used for mRNA quantification analysis. The primers for PRSS8 mRNA analysis for qRT-PCR are listed in the Supplementary Table S1.

### Cell culture

Human esophageal squamous cell carcinoma cell lines KYSE450, EC9706, TE1 and TE8, and human embryonic kidney cells HEK293 from American Type Culture Collection (ATCC) (Manassas, VA) were maintained in a complete MEM medium. All cells were free of mycoplasma contamination. All media were supplemented with 10% FBS and antibiotics (10,000 U/ml penicillin, 10 μg/ml streptomycin). Cells were cultured at 37°C in a humidified atmosphere containing 5% CO_2_.

### Methylation specific PCR

Genomic DNA from human esophageal squamous cell carcinoma tissues and ESCC cancer cells was extracted using a DNeasy Tissue Kit (Qiagen, Valencia, CA, USA). Cytosine methylation was determined using bisulfite sequencing. Briefly, DNA (up to 2 μg) was converted using DNA methylation Kit (Beijing ComWin Biotech Co.,Ltd, Bejing, China), the primers for methylation specific PCR (MS-PCR) were designed using MethPrimer [22] as showing in Supplemental Table 1. The MS-PCR products were sequenced for methylation status. The thermal cycler was programmed as follows: denaturation at 95°C for 5 min, followed by 35 cycles of denaturation at 95°C for 45 s, annealing at 56°C for 45 s and extension at 72°C for 45 s, and then 72°C for 10 min at completion. The amplified fragment for this region is 123 bp, which encompasses the 7 CpG sequences in this region. The PCR products were fractionated by electrophoresis on a 1.3% agarose gel, extracted, and cloned into a pMD 19-T vector using the TA Cloning Kit (TaKaRa, Japan). DNA sequencing was performed to verify the sequence of each ds oligo insert using pMD 19-T common primer M13F and M13R (Supplementary Table S1). At least three subclones from each sample were screened.

### Construction of truncated promoter reporters and promoter activity analysis

The genomic DNA for PRSS8 promoter reporter construction were extracted from the KYSE450 cells having hypermethylation in PRSS8 promoter, or from the TE1 having unmethylation in PRSS8 promoter region, respectively. The full length of PRSS8 promoter about 5,000 base pairs genomic fragment containing the PRSS8 cDNA start site at its 3′ end was subcloned into the Hind III and XhoI site of the luciferase reporter vector, pGL4-basic (Promega, Madison, WI, USA), to create PRSS8-5K. The proximal fragment of 2,000 base pairs of the PRSS8 promoter containing the PRSS8 cDNA start site at its 3′ end was subcloned into the Hind III and XhoI site of pGL4-basic reporter vector to construct PRSS8-2K. The distal fragment of 3,000 base pairs of the PRSS8 promoter was subcloned into the Hind III and XhoI site of pGL4-basic reporter vector to construct PRSS8-3K. The primers for these constructions were listed in Supplementary Table S1.

PRSS8 promoter activities of these truncated luciferase reporters were measured in HEK293 cells using a transient transfection assay with the co-transfection of reporter constructs and Renilla luciferase reporter. After 48 h, the cells were harvested, and firefly and Renilla luciferase activity levels were assessed using the Dual Luciferase kit (Promega, Madison, WI, USA) according to the manufacturer's specifications. The values from Renilla luciferase activity were used to correct for the differences of the transfection efficacy and promoter activities.

### Expression plasmids construction, small interfering RNA (siRNA) synthesis and transfection, and immunoblotting

The full length of PRSS8 was cloned from human cDNA and inserted into pEGFP vector (Promega, Madison, WI), to generate pEGFP -PRSS8, expression constructs. A 19-nt siRNA oligonucleotide with 3′-dt extensions against human PRSS8 transcript and one scrambled siRNA (negative control) were designed, as shown in Supplementary Table S1. siRNAs were synthesized by Shanghai GenePharma Inc. (Shanghai, China). Twenty-four hours before transfection, 1.0×10^5^ cells were seeded in a 6-well plate. 4 μg of PRSS8 expression plasmid or negative control plasmid was transfected into cells, using Lipofectamine 3000 (Invitrogen, Carlsbad, CA) following the manufacture's protocol. The cells were collected for immunoblotting analysis using the following primary antibodies: anti-PRSS8, anti-P21, anti-Cyclin D1, anti-E-cadherin, anti-snail and anti-Twist (from Cell Signaling Technologies Inc., CA). Anti-β-actin (Sigma, St Louise, MO) was used as internal loading control.

### Cell proliferation and cell cycle analysis

The MTS assay was used as a cytotoxicity assay for the KSE450 and EC9706 cells treated with 30μM or 100μM of DAC, and the cells with treatment of DAC and transfected with siR-PRSS8 using Lipofectamine 3000 (Invtrogen, Carlsbad, CA). After 24 and 48 hours, cell proliferation was determined by MTS assay (3- (4,5-dimethylthiazol-2-yl)-5-(3-carboxymethoxyphenyl)-2-(4-sulfophenyl)-2H-tetrazolium) according to the manufacturer's protocol (CellTiter 96 Non-Radioactive Cell Proliferation Assay Kit, Promega Corporation, Madison, WI). The number of viable cells with MTS uptake was determined by measuring optical density at 570 nm using an enzyme-linked immunosorbent assay reader (Molecular Devices, Sunnyvale, CA). Values shown were mean +/− standard deviation. At least three measurements were read, and the experiments were conducted 3 times independently. Changes in cell cycle were analyzed using flow cytometry with P.I. staining, as described by us recently [35].

To determine whether the restoration of PRSS8 by DAC was time-dependent, we treated the KYSE450 and EC9706 cells with 100 μM of DAC and collected the cells at 24, 48, 72, 96 and 120 hours. The cell lysate was subjected for immunoblotting.

### Cell mobility and migration assay

Cell mobility was assayed using the wound healing method. As reported previously [36], the KYSE450 and EC9706 cells were seeded in a 100-mm Petri dish, then were treated with DAC or with treatment of DAC and transfection of siR-PRSS8. A wound was made by scratching on the Petri dish bottom, followed by another 48 hours growth. Changes in the width of the wound were observed and measured under microscope.

Cell migration was analyzed by Transwell assay. In brief, the KYSE450 and EC9706 cells were seeded in transwell compartments (Corning, NY) using a 24-well format, with 8 μm pore size insert. The cells were treated with 30μM of DAC or with the treatment of DAC and transfection of siR-PRSS8. The cells in the transwell plate were incubated at 37°C and 5% CO_2_ for 24 hours. The cells on the lower side of the insert were stained with DAPI for 15 min and counted under a microscope. The same experimental procedure was performed for control group transfected with negative control plasmids. The experiments were triplicated independently.

## SUPPLEMENTARY FIGURE AND TABLE





## Abbreviations

ESCC: esophageal squamous cell carcinoma
DAC: decitabine
