# Correction: PRSS8 methylation and its significance in esophageal squamous cell carcinoma

**DOI:** 10.18632/oncotarget.17541

**Published:** 2017-05-01

**Authors:** Yonghua Bao, Qian Wang, Yongchen Guo, Zhiguo Chen, Kai Li, Yiqiong Yang, Huijuan Zhang, Huali Dong, Kui Shen, Wancai Yang

**Present**: The images of cell migration were mistakenly duplicated at Fig.5D and 5E. This error does not affect the results and conclusions in the paper or the interpretation of the data.

**Correct**: The corrected images appear below. The authors apologize for any confusion this error may have caused.

Original article: Oncotarget. 2016; 7:28540-55. doi: 10.18632/oncotarget.8677

**Figure 5:**
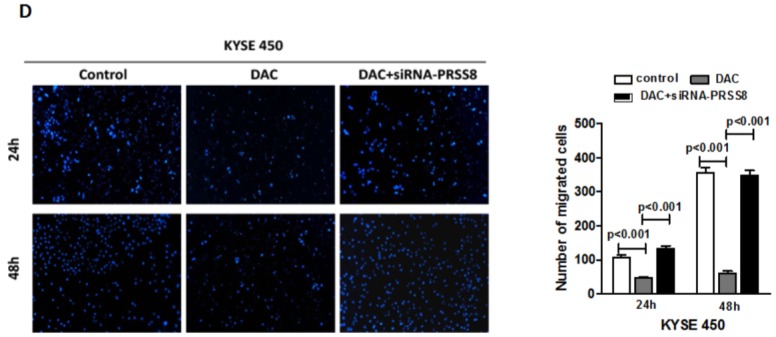

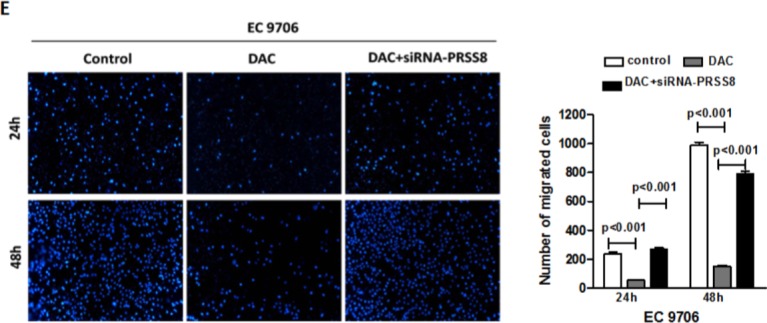
Restored PRSS8 expression by DAC led to inhibition of cell proliferation, motility and migration, but PRSS8- mediated tumor inhibition could be attenuated by small interfering RNA **A.** Restored expression of PRSS8 inhibited cell proliferation in KYSE450 and EC9706 cells, but the inhibition was reversed by siRNA targeting PRSS8 (siR-PRSS8), assayed by MTT. The statistical analysis was shown in the lower panel. **B** and **C.** Restored PRSS8 inhibited cell motility in KYSE450 cells (B) and EC9706 cells (C), but the inhibition was reversed by siRNA, assayed by wound healing. The quantification of the wound width was shown in the right panel, respectively. **C.** Restored PRSS8 inhibited cell motility in KYSE450 cells (B) and EC9706 cells (C), but the inhibition was reversed by siRNA, assayed by wound healing. The quantification of the wound width was shown in the right panel, respectively. **D.** Restored expression of PRSS8 inhibited cell migration in KYSE450 (D) and EC9706 cells (E), but the inhibition was reversed by siRNA. The number of the migrated cells was shown in the right panel, respectively. **E.** Restored expression of PRSS8 inhibited cell migration in KYSE450 (D) and EC9706 cells (E), but the inhibition was reversed by siRNA. The number of the migrated cells was shown in the right panel, respectively. **F.** The alteration of proliferation and EMT-related proteins by DAC and DAC + siR-PRSS8 in KYSE450 cells was shown. **G.** PRSS8 overexpression led to the upregulation of P21 and E-cadherin and to the downregulation of cyclin D1, Twist and Snail in KYSE450 and EC9706 cells.

