# Supplementary material for: PRSS8 methylation and its significance in esophageal squamous cell carcinoma
Source: Oncotarget. 2016 Apr 11;7(19):28540–55. doi: 10.18632/oncotarget.8677 (PMC5053744; doi:10.18632/oncotarget.8677)
Supplement: Supplementary file 1 [file oncotarget-07-28540-s001.pdf]

SUPPLEMENTARY FIGURE AND TABLE

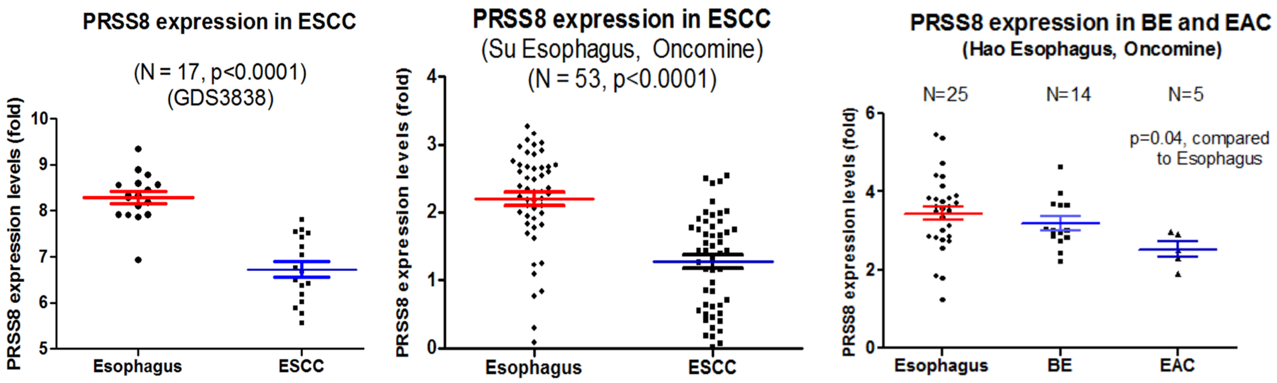

Supplementary Figure S1: PRSS8 mRNA was significantly reduced in esophageal squamous cell carcinoma (ESCC), esophageal adenocarcinoma (EAC) and Barrett's Esophagus (BE), compared to normal esophagus.

Supplementary Table S1: Primers for qRT-PCR analysis, small interfering RNA sequences, MethPrimer sequences for PRSS8 and primers for PRSS8 promoter reporter construction.

See Supplementary File 1
