# Supplementary material for: PRSS8 methylation and its significance in esophageal squamous cell carcinoma
Source: Oncotarget. 2016 Apr 11;7(19):28540–55. doi: 10.18632/oncotarget.8677 (PMC5053744; doi:10.18632/oncotarget.8677)
Supplement: Supplementary file 2 [file oncotarget-07-28540-s002.docx]

**Supplementary Table S1. Primers for qRT-PCR analysis, small interfering RNA sequences , MethPrimer sequences for PRSS8 and primers for PRSS8 promoter reporter construction.**

**Primers for qRT-PCR analysis**:

Gene name Forward primer sequence Reverse primer sequence

**_____________________________________________________________________________**

PRSS8 AGAGGACATGGTGTGTGCTG GAGGCTGGAGTTCTGTCACC

GAPDH GTCAAGGCTGAGAACGGGAA AAATGAGCCCCAGCCTTCTC

**____________________________________________________________________________**

**Small interfering RNA sequences**

Gene name-position Sense（5'-3'） Antisense（5'-3'）

_____________________________________________________________________________

PRSS8-1906 CAGGGCAGAAAUGAUUAAATT UUUAAUCAUUUCUGCCCUGTT

PRSS8-932 GUAACUGCCUGUACAACAUTT AUGUUGUACAGGCAGUUACTT

Scrambled siRNA UUCUCCGAACGUGUCACGUTT ACGUGACACGUUCGGAGAATT

**MethPrimer sequences for PRSS8**

Name Sequence

_____________________________________________________________________________

Left M primer AATAGTTGGAATTATAGGTATGCGT

Right M primer CAAACTAACAAAATAACTAATCGAC

Left U primer AATAGTTGGAATTATAGGTATGTGT

Right U primer CAAACTAACAAAATAACTAATCAAC

**Primers for PRSS8 promoter reporter construction**

Name Sense（5'-3'） Antisense（5'-3'）

_____________________________________________________________________________

2K  AT*CTCGAG* ttacccactgcttacctgca  TC*AAGCTT*ggcccaggacaagggcccct

3K AT*CTCGAG* tgggtcacgggaacgaggag TC*AAGCTT* aacctgggaagccaagattg

5K  AT*CTCGAG* tgggtcacgggaacgaggag TC*AAGCTT*ggcccaggacaagggcccct

*AAGCTT:* Hind III restriction site*; CTCGAG:*XhoI restriction site ; AT and TC: Protective base

**Sequences primers (M13F and M13R) for methylation specific PCR product**

Name Sequence

_____________________________________________________________________________

M13F(-47) 5'-d(CGCCAGGGTTTTCCCAGTCACGAC)-3'

M13R(-48) 5'-d(AGCGGATAACAATTTCACACAGGA)-3'
